# Supplementary material for: The psychosocial and economic impacts on female caregivers and families caring for children with a disability in Belu District, Indonesia
Source: PLoS One. 2020 Nov 4;15(11):e0240921. doi: 10.1371/journal.pone.0240921 (PMC7641436; doi:10.1371/journal.pone.0240921)
Supplement: S2 Fig — (DOCX) [file pone.0240921.s002.docx]

**Appendix 1: Interview Guide**

**Interview guide: mothers or female caregivers of children with a disability in Belu district, Indonesia.**

Is it OK if I record the interview?

Would you like to have someone with you while we talk?

When my colleagues and I write up the results of this study, we plan to assign a specific study identification number for you (as well as for other participants we interview) to ensure that information or comments you provide in this interview remain anonymous. Is this OK with you? You can withdraw your participation if you do not want to continue or feel uncomfortable during this interview without any consequences.

As you may have known through the study information sheet and our initial conversation once you called me to confirm your willingness to participate, the purpose of this study is to gain your insights regarding psychosocial and economic impacts of childhood disability on you and your family.

**Psychological impact:**

How do you feel about raising a child with a disability?

Does having a child with a disability cause any psychological challenges? Why? (Tell me more it).

How do you feel about the life and future of your child with a disability? (Tell me more about it)

**Social impact**:

Do you think having a child with a disability affects the relationship among family members in your family? (If any, please explain about it).

Would you mind sharing your experience about your social interaction with others within the community where you live?

Do you think caring for your child with a disability affects your social life? Why? (Please explain about it)

How is the social interaction of your child with a disability with other kids within the community where you live? (Tell me more about it)

**Economic impact**:

Would you mind sharing your experience about economic challenges you face, if any, in relation to raising your child with a disability? (Tell me more about it).

What kind expenditures in relation to caring for your child with a disability do you feel burdensome? Why? (Tell me more about it).

Do you think raising your child with a disability influences your job or career? How? (Tell me more about it).

Can I ask some questions about you?

How old are you?

Are you working at the moment? Part time or full time?

Can you tell me about your educational background?

Is there anything else you wish to add about the impacts of raising a child with a disability?

Would you like to see a copy of the interview transcript and edit it prior to us analysing the information?

Would you be able to suggest your friends who have a child with a disability and who might be willing to participate in this study?
