# Supplementary material for: The psychosocial and economic impacts on female caregivers and families caring for children with a disability in Belu District, Indonesia
Source: PLoS One. 2020 Nov 4;15(11):e0240921. doi: 10.1371/journal.pone.0240921 (PMC7641436; doi:10.1371/journal.pone.0240921)
Supplement: Attachment — Submitted filename: Response to Reviewers.docx [file pone.0240921.s003.docx]

Response to Reviewers

Dear Joseph Telfair,

Thank you very much for considering our manuscript to be published in PLOS ONE. Herewith we submit the revised version of our paper entitled “**The psychosocial and economic impacts on female caregivers and families caring for children with a disability in Belu District, Indonesia”**

On behalf of all authors,

Sincerely,

Gregorius Abanit Asa

1. Title and throughout the paper: Given the theoretical frameworks informing this study, with one heavily based on social aspects, I would encourage the authors to consider changing "psychological" to "psychosocial" impact; the study does not seem to be framed within a psychological model, but on a social and economic, thus, even the psychological impact is shaped by that. This is reflected in Theme 1 "feeling frustrated, sad and angry" and Theme 3 "Feeling inferior and insecure" of Psychological impact, which both speak about socially determined psychological states, further supporting the idea that this study teased out psychosocial impact.

**Response**:

Term psychosocial has been used throughout the manuscript.

2. The Introduction provides information about prevalence of disability in Indonesia, but no figures are presented about the proportion of children who have disabilities. This information would be useful to better contextualise the study.

**Response**:

Indonesia is one of the countries in South-East Asia region where the prevalence of people with disabilities continues to increase. The 2012 national socioeconomic survey reported that the percentage of people with a disability in the country increased from 1.38% in 2006 to 2.45% in 2011 (4). In 2017 same survey reported over 37.1 million people live with a disability, and about 10% ( 3.2 million) are children(5).

3. The intro justifies researching the economic impact of caregiving due to a gap in the knowledge, but there is no evident rationale for investigating the psychosocial impact, and the literature discussed in the Introduction appears to be quite extensive on this topic. It would be useful to clarify for the reader why psychosocial impacts were investigated.

**Response**:

In addition, psychosocial impacts were also explored to add new knowledge and to provide a comprehensive understanding of the lived experiences and impacts of childhood disability on health and general wellbeing of mothers or female caregivers in this study and in Indonesia where evidence on the impacts of childhood disability is still limited (41, 42).

4. Study Setting: The authors provide a clear description of Belu, but it would be good to explain briefly why this setting was chosen. Are there differences in prevalence of children with disabilities between this district and the national context?

**Response**:

Belu is reported as one of the districts in East Nusa Tenggara province with the highest number of children living with a disability, accounting 348 children (50). To the best of our knowledge, there is also limited research evidence illuminating the impacts of childhood disability on parents or caregivers in Indonesia(41, 42). Because of small size, feasibility, familiarity and the potential of undertaking the current study successfully, Belu was selected as the study setting.

5. Study design and data collection: It would be useful to have more information about the interviews: were they structured or semi-structured? What was the range of duration of the interview? I would strongly encourage the authors to add the interview schedule or main interview questions in a supplementary file. Also, how were questions decided and by which members of the research team? What equipment was used to record the interviews?

**Response**:

- The interviews were conducted by the first author (male) who is a freelance researcher and have attended a formal training in qualitative methods during master’s study. Interviews were guided by several predetermined main questions and probing questions were developed alongside the progress of each interview to allow the participants to communicate much more freely and to provide more detailed descriptions or information about the topics being investigated (54-56). The decision about the questions was made through a process of formulation, discussion and revision by the first and second authors, and based on the conceptual frameworks used in this study and the study aim (57).
- The interviews were carried out in Bahasa with the duration ranging between 30 to 45 minutes and recorded using a digital recorder. Notes were also taken during the interviews.
- Main interview questions are submitted as a supplementary file.

6. Study Design: The main body of the manuscript does not make reference to the COREQ checklist in the supplementary file, which would be important to mention. Importantly, the COREQ checklist should include the page number where each point of the list has been addressed, which has not been done in the submitted document. I also note that some points of the checklist are ticked but have not been addressed, as for instance point 19 on audio/video recording.

**Response**:

- COREQ checklist has been referenced in the manuscript.
- Consolidated criteria for reporting qualitative studies (COREQ) checklist (45) was used to guide the report of the methods section of this study. The COREQ checklist contains 32 (S1 Fig.) required items for explicit and comprehensive reporting of qualitative studies especially interviews and focus groups (45).
- COREQ checklist with page number has been provided as a supplementary file and each item in the checklist has been addressed in the manuscript.

7. Results: While I agree with the authors about describing the psychological impact separately from the social and economic impact, I would suggest to move this as the final theme, because there are important overlaps between the three themes and the psychological wellbeing of the participants appears to be a result of the social and economic impact. Just a suggestion.

**Response**:

Psychological impact has been moved as the final theme.

8. The Discussion is overall clear, but I feel that the summary of main findings could be less descriptive and try to go a bit deeper into the mechanisms leading to the psychosocial and financial impact considering the particular social and cultural context. Do we see here an accumulation of social stigma and financial strains that are found in other regions? How much of the findings can be generalised? These aspects are touched upon, but the summary of findings is very long, and the more speculative part is a bit lost.

**Response**:

Revisions have been made to the discussion section.

9. The Conclusions section suggests potential interventions, but it doesn't appear to touch on implications for tackling social/cultural attitudes and stigma. As they say, 'it takes a village', and this study clearly highlights how these women live in communities where they don't feel supported emotionally, psychologically, let alone financially. This is an important point emerging from this study, I feel that it should be given more weight.

**Response**:

Similarly, findings also indicate the need for disability-related awareness raising and education for family and community members in Belu and other settings in the country to increase their knowledge and awareness of disability and acceptance of and support for people with a disability people, which may reduce stigma and discrimination against children with a disability and their family.
